# Striatal dopaminergic patterns and clinical features in frontotemporal dementia

**DOI:** 10.1093/braincomms/fcaf284

**Published:** 2025-08-20

**Authors:** Daniele Urso, Antonio Anastasia, Valentina Gnoni, Alessia Giugno, Davide Vilella, Alessandra Vitulli, Chiara Zecca, José A Pineda-Pardo, Guglielmo Foffani, José A Obeso, Giancarlo Logroscino

**Affiliations:** Center for Neurodegenerative Diseases and the Aging Brain, Department of Clinical Research in Neurology, University of Bari ‘Aldo Moro’, 73039 Tricase, Lecce, Italy; Center for Neurodegenerative Diseases and the Aging Brain, Department of Clinical Research in Neurology, University of Bari ‘Aldo Moro’, 73039 Tricase, Lecce, Italy; Department of Nuclear Medicine, Pia Fondazione di Culto e Religione ‘Card.G.Panico’, 73039 Tricase, Italy; Center for Neurodegenerative Diseases and the Aging Brain, Department of Clinical Research in Neurology, University of Bari ‘Aldo Moro’, 73039 Tricase, Lecce, Italy; Center for Neurodegenerative Diseases and the Aging Brain, Department of Clinical Research in Neurology, University of Bari ‘Aldo Moro’, 73039 Tricase, Lecce, Italy; Center for Neurodegenerative Diseases and the Aging Brain, Department of Clinical Research in Neurology, University of Bari ‘Aldo Moro’, 73039 Tricase, Lecce, Italy; Center for Neurodegenerative Diseases and the Aging Brain, Department of Clinical Research in Neurology, University of Bari ‘Aldo Moro’, 73039 Tricase, Lecce, Italy; Center for Neurodegenerative Diseases and the Aging Brain, Department of Clinical Research in Neurology, University of Bari ‘Aldo Moro’, 73039 Tricase, Lecce, Italy; HM CINAC (Centro Integral de Neurociencias Abarca Campal), Hospital Universitario HM Puerta del Sur, HM Hospitales, 28938 Madrid, Spain; CIBERNED, Instituto de Salud Carlos III, 28029 Madrid, Spain; HM CINAC (Centro Integral de Neurociencias Abarca Campal), Hospital Universitario HM Puerta del Sur, HM Hospitales, 28938 Madrid, Spain; CIBERNED, Instituto de Salud Carlos III, 28029 Madrid, Spain; HM CINAC (Centro Integral de Neurociencias Abarca Campal), Hospital Universitario HM Puerta del Sur, HM Hospitales, 28938 Madrid, Spain; CIBERNED, Instituto de Salud Carlos III, 28029 Madrid, Spain; Center for Neurodegenerative Diseases and the Aging Brain, Department of Clinical Research in Neurology, University of Bari ‘Aldo Moro’, 73039 Tricase, Lecce, Italy; Department of Translational Biomedicine and Neurosciences (DiBraiN), University of Bari Aldo Moro, 70124 Bari, Italy

**Keywords:** frontotemporal dementia, primary progressive aphasia, dopaminergic, striatum, imaging

## Abstract

Frontotemporal dementia is a group of neurodegenerative disorders mainly characterized by behavioural and language impairments. While the precise pathophysiology remains elusive, emerging evidence points to an important role of dopamine dysfunction, particularly within the caudate nucleus. Moreover, a theoretical model proposes that frontotemporal dementia manifestations result from a deficit in goal-directed behaviour, which may be related to altered dopamine control of the frontostriatal circuitry. However, no study has investigated the gradient of striatal dopamine transporter levels in frontotemporal dementia using neuroimaging and their correlation with clinical features. This study used ^123^I-Ioflupane Single Photon Emission Computed Tomography imaging to measure striatal dopamine transporter levels and their distribution patterns in frontotemporal dementia, compared to Parkinson’s disease and healthy controls. Additionally, we explored the correlation between dopamine transporter uptake and two key domains affected in frontotemporal dementia: social cognition and language abilities. We hypothesized that frontotemporal dementia would show a predominant dopaminergic deficit in the caudate, and that this would correlate with the severity of clinical core features. The study comprised 139 participants, including 34 sporadic and genetic frontotemporal dementia, 68 Parkinson’s disease individuals, and 37 age- and sex-matched healthy controls. Among the frontotemporal dementia group, 22 cases had clinically probable behavioural variant frontotemporal dementia, and 12 had primary progressive aphasia. Social cognition was assessed with the abbreviated version of the Social and Emotional Assessment, which includes a Theory of Mind test and a Facial Emotion Recognition Task. Language skills were evaluated with the Screening for Aphasia in NeuroDegeneration battery. We found that dopamine transporter levels were reduced in frontotemporal dementia compared to healthy controls (*P* < 0.001) and that frontotemporal dementia showed a higher putamen-to-caudate ratio than Parkinson’s disease (*P* < 0.001), particularly notable in patients with identified disease-causing mutation. We also found that dopamine transporter levels were correlated with parkinsonian motor features and general cognition in frontotemporal dementia. Notably, both social cognition—especially facial emotion recognition—and language abilities exhibited associations with dopamine transporter levels in both the putamen and the caudate. These findings suggest that the pattern of dopamine transporter uptake could serve as a valuable biomarker for frontotemporal dementia, shedding light on the role of the dopaminergic system and the striatum in some fundamental clinical aspects. This opens new avenues for further investigating the underlying mechanisms and potential therapeutic targets of the dopaminergic projections in frontotemporal dementia.

## Teaser

Urso *et al*. report that striatal dopamine transporter levels are reduced in frontotemporal dementia, with both the putamen and caudate similarly affected. These reductions correlate with core clinical features of frontotemporal dementia, including social cognition and language, suggesting that dopaminergic imaging may aid in the diagnosis and disease characterization.

## Graphical Abstract

**Graphical Abstract:**
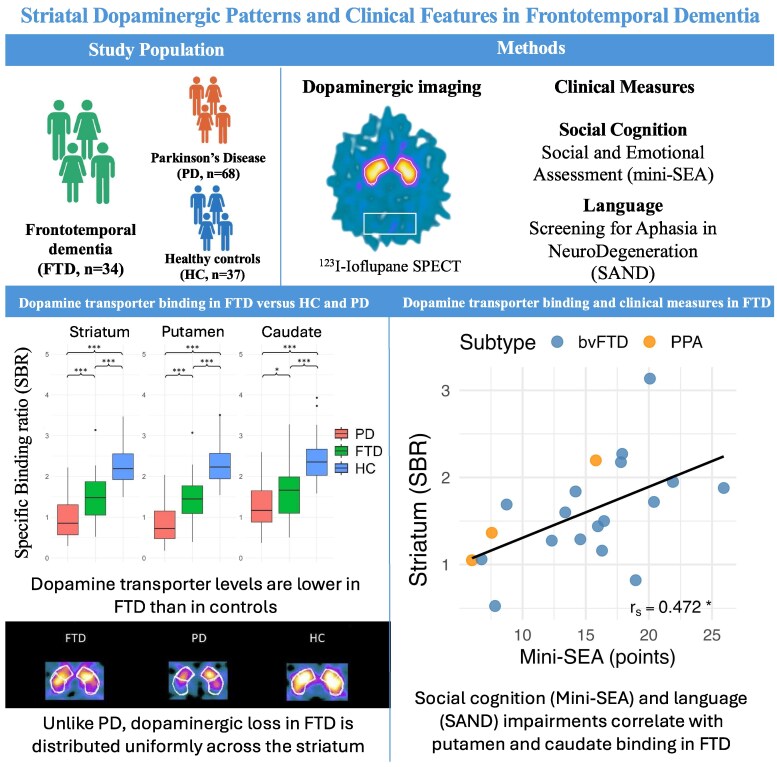

## Introduction

Frontotemporal dementia (FTD) encompasses a group of neurodegenerative disorders that lead to diverse and insidious clinical syndromes, mainly characterized by behavioural changes and language impairments.^1^ It is often misdiagnosed or diagnosed late, due to the lack of specific biomarkers and the overlap with other neurological conditions.^2^ The most common clinical subtypes of FTD are the behavioural variant of frontotemporal dementia (bvFTD) and two types of primary progressive aphasia (PPA): semantic and non-fluent. bvFTD, the most prevalent type of frontotemporal dementia, is fundamentally a disorder of social cognition.^3^ It manifests with a variety of symptoms, such as disinhibited behaviour, apathy, heightened consumption of sweet foods and alcohol, diminished empathy and emotional processing, and compromised executive function.^4^ Semantic dementia, on the other hand, is marked by a deterioration of semantic knowledge, typically presenting as progressive anomia, within the framework of fluent expressive speech.^5^ In contrast, non-fluent PPA is characterized by laborious and distorted speech, with or without agrammatism, while single-word comprehension remains intact.^5^ bvFTD is the second most common form of younger-onset dementia after Alzheimer’s disease, frequently occurring before 65 years of age. However, recent studies have found that FTD clinical syndromes are more common than previously described, and their diagnosis needs to be considered also in the elderly, beyond the age of 70 years.^6-8^

Clinical and experimental observations point towards a dopamine deficit in FTD, characterized by loss of pre-synaptic dopaminergic neurons, abnormal dopamine receptor binding, reduced dopamine transporter binding, and reduced dopamine levels.^9^ In fact, reduced dopamine levels have been detected using high-performance liquid chromatography in areas such as the putamen, caudate, and substantia nigra.^10,11^ Dopamine D2 receptors are reduced in the frontal lobes of patients with FTD,^12^ while reduced levels of dopamine and its metabolites in the cerebrospinal fluid (CSF) have also been reported in FTD,^13^ which correlates with agitation and caregiver burden.^14^ Recently, a framework has been proposed that explains many of the clinical manifestations observed in FTD as a consequence of a disturbed goal-directed behaviour,^15^ leading to a shift towards habitual patterns of behaviour. Striatal dopaminergic activity is known to control/modulate both goal-directed and habitual behaviours.^16^  *In vivo* imaging studies have demonstrated a reduction in dopamine transporter levels—a marker indicating degeneration of nigrostriatal dopaminergic pathways—in the caudate and putamen of patients with FTD.^17-19^ Moreover, the extent of this reduction correlates with the severity of parkinsonian features,^17,18^ which are highly prevalent in this neurodegenerative disease.^20^ Similarly, diagnostic accuracy study demonstrated that an abnormal ^123^I-Ioflupane Single Photon Emission Computed Tomography (SPECT) scan, even in patients with parkinsonian features, does not exclude the diagnosis of FTD.^21^ Pathology and imaging studies have highlighted the frequent occurrence of neuronal loss in the striatum among FTD patients is frequent,^22-26^ which precedes focal cortical atrophy of behavioural and language networks.^27^ This suggests that striatal atrophy could potentially serve as a biomarker for defining preclinical or prodromal FTD variants.^27^ Moreover, some recent case reports have suggested that dopamine transporter levels are decreased, especially in the caudate,^28-31^ suggesting a striatal degeneration pattern in FTD that differs from the typical posterior-to-anterior gradient observed in Parkinson’s disease (PD).^32^

Despite these previous findings, no study has yet systematically evaluated the patterns of nigrostriatal degeneration in FTD and their correlation with cardinal clinical features. Therefore, in this study we use ^123^I-Ioflupane SPECT, a widely available dopamine transporter imaging modality, to (i) assess dopamine transporter levels in the striatum of patients with FTD, compared with PD and healthy controls, (ii) assess the putamen-to-caudate ratio, reflecting the distribution pattern of striatal dopaminergic loss in FTD, compared with PD and healthy controls, and (iii) explore whether dopamine transporter levels in the striatum are associated with cardinal clinical manifestations of FTD, namely social cognition and language. We hypothesize that dopaminergic deficit in FTD is predominantly in the caudate, and dopaminergic striatal degeneration correlates with the principal FTD clinical features.

## Materials and methods

### Ethical approval

The local Medical Ethics Committee approved the protocol for using the clinical data for research purposes (Protocol No: 6, 25 July 2017).

### Study participants

Thirty-eight patients with a clinical diagnosis of behavioural variant frontotemporal dementia (bvFTD) or primary progressive aphasia (PPA), whose amyloid status data were available, diagnosed between January 2021 and September 2023, were prospectively recruited at the Center for Neurodegenerative Diseases and the Aging Brain of the University of Bari Aldo Moro at Pia Fondazione ‘Card. G. Panico’. All patients underwent an extensive assessment, including medical examination, neuropsychological tests, language assessment, and imaging (3T MRI or CT scan) and a lumbar puncture for CSF biomarkers analysis, as part of the diagnostic procedure. All patients were diagnosed by a multidisciplinary team according to clinical diagnostic criteria for probable bvFTD^4^ or PPA.^5^ Four patients who had a positive Alzheimer’s disease CSF profile and/or a positive amyloid-PET scan were excluded. In addition, all participants with clinical features suggestive of progressive supranuclear palsy, such as early postural instability, unexplained falls, or vertical gaze palsy, were excluded. The final sample comprised 22 subjects with bvFTD and 12 subjects with PPA (eight non-fluent/agrammatic variant, three semantic variant and one mixed variant^33^) and were included in the analysis. Seven of the FTD subjects had an identified disease-causing mutation (four *C9orf72*, three *GRN*). All of these cases had a bvFTD clinical syndrome except for one that was a PPA. All FTD individuals underwent dopamine transporter imaging to measure dopamine transporter binding using ^123^I-Ioflupane SPECT imaging as part of this study. To compare DAT binding, age and sex-matched Parkinson’ disease (*n* = 68) patients diagnosed between January 2021 and September 2023 were included in this study. PD diagnosis was made according to the latest Movement Disorders Society diagnostic criteria for clinically established PD.^34^ All patients underwent dopamine transporter imaging to measure dopamine transporter binding using ^123^I-Ioflupane SPECT imaging. We excluded patients with normal DAT binding^34^ and any clinically significant neurological disorders. Additionally, 37 age- and sex-matched healthy controls (HC) were added as a reference for dopamine transporter binding. Data used for HC were obtained from the Parkinson’s Progression Markers Initiative database (www.ppmi-info.org/data) in September 2023. HC were eligible if they had no significant neurological or psychiatric disorders, Montreal Cognitive Assessment (MoCA) scores above 26, and no first-degree relatives with PD, and if they showed normal DAT binding. None of the patients or control subjects received dopaminergic medication or medication known to affect dopaminergic function,^21^ including alpha methyldopa, methylphenidate, amphetamine derivatives, or modafinil. No participant had a significant burden of cerebrovascular disease or visual loss. This study was approved by the Institutional Review Board of ASL Lecce (verbale n. 6, 25 July 2017), and all participants gave informed consent in accordance with the Declaration of Helsinki.

### Clinical data collection and assessment

Disease duration for each patient was calculated from the time of symptom onset to the date of enrolment in the study. Symptom onset was defined as the point when the patient or informant first noticed significant cognitive or behavioural changes for bvFTD, language problems for PPA, and motor problems for PD. Parkinsonian motor features were systematically assessed using the motor part (Part III) of the MDS Unified Parkinson’s Disease Rating Scale (MDS-UPDRS)^35^ by a trained neurologist experienced in movement disorders (D.U). The severity of functional impairment in patients was evaluated using the Clinical Dementia Rating scale (CDR).^36^ Patients with a CDR global score greater than 2, indicative of more than moderate dementia severity, were excluded from the study as their cognitive and functional impairment might have interfered with the accuracy of assessments. Cognitive function was assessed using the Mini-Mental State Examination (MMSE)^37^ and the Montreal Cognitive Assessment (MoCA).^38^ All assessments, including social cognition and language assessments described below, were conducted by trained neuropsychologists and clinicians who were blinded to the patients’ dopamine transporter imaging results. The assessments were performed within a close timeframe to minimize variability due to disease progression or other factors.

### Social cognition assessment

Seventeen patients diagnosed with bvFTD and four individuals with PPA underwent the abbreviated version of the Social and Emotional Assessment (Mini-SEA),^39^ which were validated in a previous study, to be able to accurately discriminate bvFTD from controls or patients with Alzheimer’s disease (AD).^40^ This comprehensive evaluation comprises a Theory of Mind (ToM) test known as the Faux-Pas Test and a Facial Emotion Recognition Task. For the assessment of Theory of Mind (ToM), we utilized a concise version consisting of ten stories adapted from the Faux-Pas Test^41^ to gauge individuals’ ability to comprehend social situations. The Faux-Pas Recognition Test contains ten previously published narratives, with five describing a ‘faux-pas’ scenario (characterized by inadvertently saying something inappropriate) and five without such instances. Each ‘faux-pas’ story is rated out of 6, while stories without ‘faux-pas’ are scored out of 2. The cumulative score on the Faux-Pas Test, with a maximum of 40, is determined by adding the subscores from ‘faux-pas’ stories (five stories × 6 points = 30) and the subscores from stories without ‘faux-pas’ (five stories × 2 points = 10). Control question scores are not included in the total Faux-Pas Test score. Additionally, participants completed the Facial Emotion Recognition Task, which involved identifying the emotion expressed in 35 facial images from Ekman’s collection.^42^ The images, shown five times each during the test, represented seven emotions: fear, sadness, disgust, surprise, anger, happiness, and neutrality. A general recognition percentage was computed based on the number of correct responses.

The scores on both the Faux-Pas Test and the Facial Emotion Recognition Task were standardized to a scale of 15 for ease of comparison.^40^ The composite Mini-SEA score (/30) was obtained by summing the Facial Emotion Recognition Task (/15) and Faux-Pas Test (/15) scores, where lower scores indicate a higher degree of impairment. This comprehensive evaluation typically takes approximately 30 min to complete. For detailed test designs, instructions, and scoring methods, refer to a previously published study.^40^

### Language assessment

The Screening for Aphasia in NeuroDegeneration (SAND)^43^ battery was used to evaluate the language skills of all patients with PPA and 17 individuals with bvFTD. The SAND is a screening tool that can identify the main linguistic features needed for the diagnosis and classification of PPA. It consists of nine tests: naming pictures, understanding sentences, comprehending single words, repeating words and non-words, repeating sentences, reading, writing, making semantic associations and describing pictures. For more information on the test structure, instructions and scoring methods, please refer to the Supplemental Materials.

### ^123^I-Ioflupane SPECT and processing

Each individual received a bolus intravenous injection of ^123^I-Ioflupane (GE Healthcare Ltd) with a mean activity of 185 MBq. SPECT images for all subjects were obtained using the same dual-headed gamma camera (Philips Precedence) at 180-min post-injection, following the European Association of Nuclear Medicine (EANM) guidelines.^44^ A nuclear medicine physician with specific expertise in dopaminergic imaging (A.A.) verified the quality of the images. DaTQUANT™ software (GE Healthcare) was used for the semi-quantitative analysis of DAT-SPECT images in all three groups of study participants. Striatal regions of interest (ROIs) were automatically delineated by the software after image reorientation and normalization to a standard brain template in Montreal Neurological Institute (MNI) space. Standardized, predefined volumetric templates were applied to identify bilateral caudate, putamen, and occipital reference ROIs, ensuring consistent anatomical positioning across subjects. Specific binding ratios (SBRs) for the right, left, and bilateral putamen and caudate were calculated as the ratio of mean counts in the target striatal ROI to the mean counts in the occipital reference ROI, reflecting dopamine transporter availability.^45,46^

### Putamen-to-caudate ratio and asymmetry in ^123^I-Ioflupane SPECT

Putamen-to-caudate ratios and striatal asymmetry were calculated and used in statistical analyses. Putamen-to-caudate ratios were obtained by dividing the specific binding ratio (SBR) in the putamen by the SBR in the caudate. Values lower than one of the putamen-to-caudate ratio indicate lower levels of DaT binding in the putamen relative to the caudate nucleus, while values higher than one indicate lower levels of DaT binding in the caudate relative to the putamen nucleus. This index was used to differentiate idiopathic Parkinson’s disease (PD) from atypical Parkinsonism,^47^ vascular Parkinsonism,^48^ and traumatic brain injury (TBI).^49^

Asymmetry striatal indices were calculated, based on previous studies,^50,51^ as follows:


$$\frac{|SBR_{\mathrm{left}}-SBR_{\mathrm{right}}|}{\frac{SBR_{\mathrm{left}}+SBR_{\mathrm{right}}}{2}}\times 100$$

Higher value of the asymmetry index indicates a greater difference in DaT binding between the two sides of the striatum. Striatal asymmetry indices were found to be significantly lower in patients with vascular parkinsonism compared to idiopathic PD.^52^

### Statistical analysis

Descriptive statistics were computed by using median (IQR) or frequencies, as appropriate. We tested the normality of all continuous data using the Shapiro–Wilk test. We compared categorical variables between groups (Frontotemporal Dementia, Parkinson’s Disease, or Healthy controls) using chi-square tests, and continuous variables using Kruskal–Wallis tests, as the data were not normally distributed. We performed *post hoc* comparisons with the Mann–Whitney U-test, adjusting for multiple comparisons with the Bonferroni correction. We compared categorical and continuous variables between subgroups (behavioural variant Frontotemporal Dementia and Primary progressive aphasia) using chi-square and Mann–Whitney tests, respectively. We reported the inferential statistics as test statistic, and *P*-value for the chi-square, Kruskal–Wallis tests and Mann–Whitney tests. The primary outcome was defined as the group comparison of the putamen-to-caudate ratio. This outcome was prioritized based on findings from an interim analysis, conducted midway through the study. To evaluate the diagnostic performance of striatal SBR measures, we conducted receiver operating characteristic (ROC) curve analyses comparing FTD against PD and HC. We computed the area under the curve (AUC) for the striatum, putamen, and caudate to quantify the discriminatory accuracy of these measures. For the exploratory correlation analysis between regional specific binding ratios of ^123^I-Ioflupane SPECT and clinical measures, we used partial Spearman’s rank correlation coefficient, controlling for age and sex. We reported the correlation coefficients as Spearman’s rho and *P*-value. Additional analyses evaluating the representativeness of the control group included in the present study are provided in the Supplementary Materials. The statistical analyses were performed using R studio 23.03.1 (http://www.rstudio.com/) with R 4.3.0. We set the significance level at a corrected *P*-value of <0.05.

## Results

### Demographic and clinical information

The participant groups did not exhibit significant differences in age (*H* = 4.7*, P* = 0.096, **Table 1**) or sex distribution (χ^2^ = 0.1, *P* = 0.93), indicating a well-matched sample across groups. However, the duration of disease was notably longer in individuals with Parkinson's disease (PD) compared to those with Frontotemporal Dementia (FTD) (*U* = 4.9, *P* = 0.026). Expectedly, FTD participants displayed higher scores in the CDR Global score (*U* = 76.3, *P* < 0.001) and lower scores in the MMSE (*U* = 11.2, *P* < 0.001) compared to other groups. Furthermore, the MoCA scores were lower in FTD compared to HC (*U* = 39.1, *P* < 0.001). Evaluating parkinsonian features using the MDS-UPDRS revealed significant differences among groups (*H* = 141.4, *P* < 0.001). On average, PD patients exhibited a mean MDS-UPDRS score of 30.59 (SD, 12.65), indicating a higher burden of motor features compared to HC and FTD. Individuals with FTD showed an average MDS-UPDRS score of 13.97 (SD, 13.92), indicating a higher burden of motor features than HC.

**Table 1:** Demographic and clinical and ^123^I-Ioflupane SPECT characteristics of frontotemporal dementia, Parkinson’s disease patients and healthy controls

	Frontotemporal dementia	Parkinson’s disease	Healthy controls	Test statistic
Demographics				
* n*	34	68	37	
Age, years	66.56 (9.65)	68.65 (10.95)	66.52 (5.34)	*H* = 4.7, *P* *=* 0.096
Sex (male/female)	23/11	48/20	25/12	χ2 = 0.1, *P* = 0.93
Disease Duration, years	3.65 (2.27)^a^	5.25 (3.90)^c^	NA	*U* = 4.9, *P* = 0.026
Global				
CDR Global score	1.11 (0.58)^a^	0.04 (0.17)^c^	NA	*U* = 76.3, ***P*** **<** **0.001**
MMSE	21.50 (6.62)^a^	25.60 (4.14)^c^	NA	*U* = 11.2, ***P*=<0.001**
MoCA	17.35 (6.06)	NA	28.38 (1.04)	*U* = 39.1, ***P*=<0.001**
MDS-UPDRS III	13.97 (13.92)^a,b^	30.59 (12.65)^a^	1.05 (1.47)	*H* = 88.5, ***P*=<0.001**
Specific binding ratios				
Mean striatum	1.49 (0.58)^a,b^	0.96 (0.49)^a^	2.27 (0.47)	*H* = 73.6***, P*=<0.001**
Mean putamen	1.44 (0.57)^a,b^	0.84 (0.45)^a^	2.30 (0.48)	*H* = 82.1, ***P*=<0.001**
Mean caudate	1.58 (0.60)^a,b^	1.25 (0.56)^a^	2.39 (0.54)	*H* = 58.2, ***P*=<0.001**

Mean (standard deviation) scores are shown unless otherwise indicated. CDR, Clinical Dementia Rating Scale; MMSE, Mini-Mental State Examination; MoCA, Montreal Cognitive Assessment; MDS-UPDRS, Movement Disorders Society Unified Parkinson’s Disease Rating Scale. Specific Binding Ratios refers to 123I-Ioflupane SPECT specific bindings in the striatum, putamen and caudate. Bolded *P*-values indicate statistically significant group differences.

^a^Different from healthy controls at *P* < 0.05;.

^b^Different from Parkinson’s Disease at *P* < 0.05;.

### Dopamine transporter binding with ^123^I-Ioflupane SPECT

**Figure 1:**
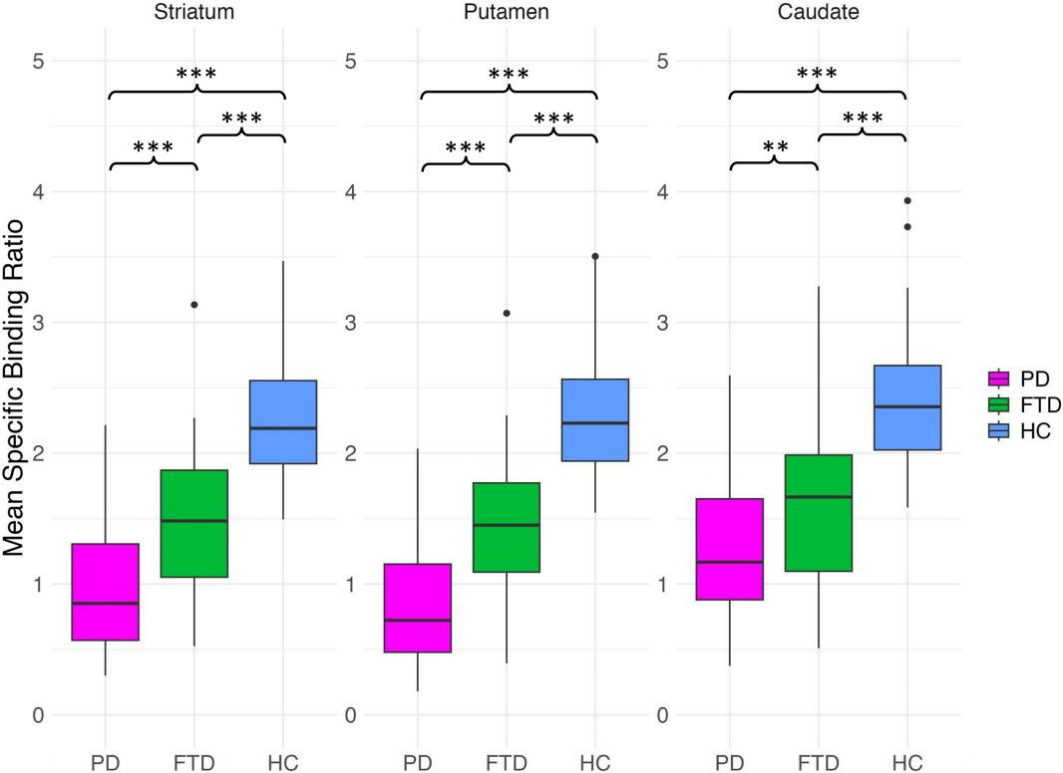
**Dopamine transporter binding in FTD, PD and controls.** Box and whisker plots displaying the distribution of specific binding ratios of ^123^I-Ioflupane among the Parkinson’s Disease (PD, n = 68), Frontotemporal Dementia (FTD, *n* = 34), and Healthy Controls (HC, *n* = 37) groups. The box represents the interquartile range (IQR; 25–75th percentile) with the median value (horizontal line) inside. The whiskers extend from the box to the minimum and maximum values that are not outliers, and the outliers are shown as dots. Outliers are defined as values outside the range [Q1–1.5 * IQR, Q3 + 1.5 * IQR], where Q1 is the 25th percentile and Q3 is the 75th percentile. Statistical analysis was conducted using the Kruskal–Wallis test for group comparison (striatum: H = 73.6; putamen: H = 82.1; caudate: H = 58.2; all *P* < 0.001), followed by Mann–Whitney U-tests with Bonferroni correction for post hoc pairwise comparisons. *P-value < 0.05, *** *P*-value < 0.001.

Dopamine transporter binding in the whole striatum, putamen and the caudate separately showed significant differences between groups (striatum *H* = 73.6, putamen *H* = 82.1, caudate *H* = 58.2; *P* < 0.001, **Table 1**, Fig. 1). *Post hoc* tests (Mann–Whitney U-tests corrected for multiple comparisons with Bonferroni adjustment) revealed that there were significant differences between all groups for each variable. Specifically, FTD and PD had lower dopamine transporter binding than HC in the striatum, the putamen, and the caudate (all *P* < 0.001). PD had lower dopamine transporter binding than FTD in the striatum, the putamen (both *P* < 0.001), and the caudate (*P* = 0.040). In differentiating FTD from HC, dopamine transporter binding measures showed good overall diagnostic accuracy, with AUCs of 0.886 for the putamen, 0.858 for the striatum, and 0.854 for the caudate. In contrast, the ability to distinguish FTD from PD was lower, with AUCs of 0.796 for the putamen, 0.761 for the striatum, and 0.662 for the caudate (Supplementary Fig. 1).

### Putamen-to-caudate ratio and asymmetry index

**Figure 2:**
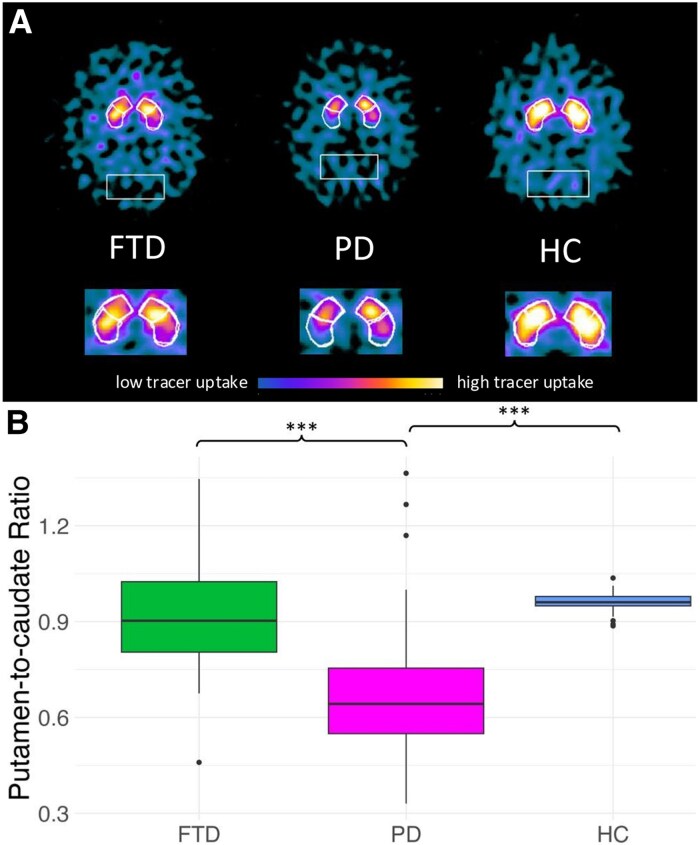
**Gradient of dopamine transporter binding in FTD.** Exemplary axial views showing predefined striatal volumes of interest, comparing tracer uptake in a patient with Frontotemporal Dementia (FTD), a patient with Parkinson’s disease (PD), and a healthy control (HC) subject (**A**). The reference VOIs are set to the bilateral occipital lobes. While the patient with PD exhibits a more significantly reduced signal posteriorly, the patient with FTD displays a more heterogeneous signal reduction, distributed both anteriorly and posteriorly. Box and whisker plots displaying the distribution of putamen-to-caudate ratio among the Parkinson’s disease (PD, *n* = 68), frontotemporal dementia (FTD, *n* = 34), and healthy controls (HC, *n* = 37) groups (**B**). The box represents the interquartile range (IQR; 25–75th percentile) with the median value (horizontal line) inside. The whiskers extend from the box to the minimum and maximum values that are not outliers, and the outliers are shown as dots. The putamen-to-caudate ratio provides an index of the posterior-to-anterior dopaminergic loss gradient; lower values of the putamen-to-caudate ratio indicate lower levels of DaT binding in the putamen relative to the caudate nucleus, while higher values indicate lower levels of DaT binding in the caudate relative to the putamen. Statistical analysis was performed using the Kruskal–Wallis test (*H* = 62.5, *P* < 0.001) followed by Mann–Whitney U-tests with Bonferroni correction. *** *P*-value < 0.001.

The putamen-to-caudate ratio was significantly different among groups (*H* = 62.5; *P* < 0.001, **Table 2,** Fig. 2). *Post hoc* analysis revealed that FTD had a higher putamen-to-caudate ratio than PD (*P* < 0.001), and that PD had a lower putamen-to-caudate ratio than HC (*P* < 0.001). This indicates that FTD have higher levels of dopamine transporter binding in the putamen relative to the caudate nucleus, when compared with PD. The striatal asymmetry index was significantly different among groups (*H* = 41.0; *P* < 0.001). *Post hoc* analysis showed that FTD and PD (both *P* < 0.001) had a higher asymmetry index than HC and that PD (both *P* < 0.001) had a higher asymmetry index than FTD (*P* < 0.001).

**Table 2:** Differences in regional specific binding ratios of ^123^I-Ioflupane SPECT and composite measures of dopaminergic striatal imaging in patients with frontotemporal dementia, Parkinson’s disease patients and healthy controls

	Frontotemporal Dementia	Parkinson’s Disease	Healthy Controls	Test statistic
Specific binding ratios				
Right striatum	1.44 (0.57)^a,b^	0.93 (0.52)^a^	2.26 (0.49)	*H* = 71.8, ***P*** **<** **0.001**
Left striatum	1.54 (0.62)^a,b^	0.99 (0.48)^a^	2.27 (0.47)	*H* = 73.7, ***P*** **<** **0.001**
Right putamen	1.40 (0.56)^a,b^	0.85 (0.47)^a^	2.21 (0.48)	*H* = 76.8, ***P*** **<** **0.001**
Left putamen	1.47 (0.62 ^a,b^	0.84 (0.46)^a^	2.20 (0.43)	*H* = 79.9, ***P*** **<** **0.001**
Right caudate	1.53 (0.57)^a,b^	1.22 (0.59)^a^	2.38 (0.55)	*H* = 56.9, ***P*** **<** **0.001**
Left Caudate	1.63 (0.67)^a,b^	1.27 (0.56)^a^	2.41 (0.54)	*H* = 56.1, ***P*** **<** **0.001**
Composite measures				
Striatal asymmetry index	15.85 (21.45)^a^	24.72 (32.57)^a^	3.70 (3.01)	*H* = 41.0, ***P*** **<** **0.001**
Putamen-to-caudate ratio	0.92 (0.19)^,b^	0.68 (0.20)^a^	0.96 (0.03)	*H* = 62.5, ***P*** **<** **0.001**

Mean (standard deviation) scores are shown unless otherwise indicated; The putamen-to-caudate ratio provides an index of the posterior-to-anterior dopaminergic loss gradient. Lower values of the putamen-to-caudate ratio indicate lower levels of DaT binding in the putamen relative to the caudate nucleus, while higher values indicate lower levels of DaT binding in the caudate relative to the putamen nucleus. Higher value of the asymmetry index indicates a greater difference in DaT binding between the two sides of the striatum. Bolded *P*-values indicate statistically significant group differences.

^a^Different from healthy controls at *P* < 0.05;.

^b^Different from Parkinson’s Disease at *P* < 0.05;.

### Dopamine transporter binding and clinical measures in FTD

**Figure 3:**
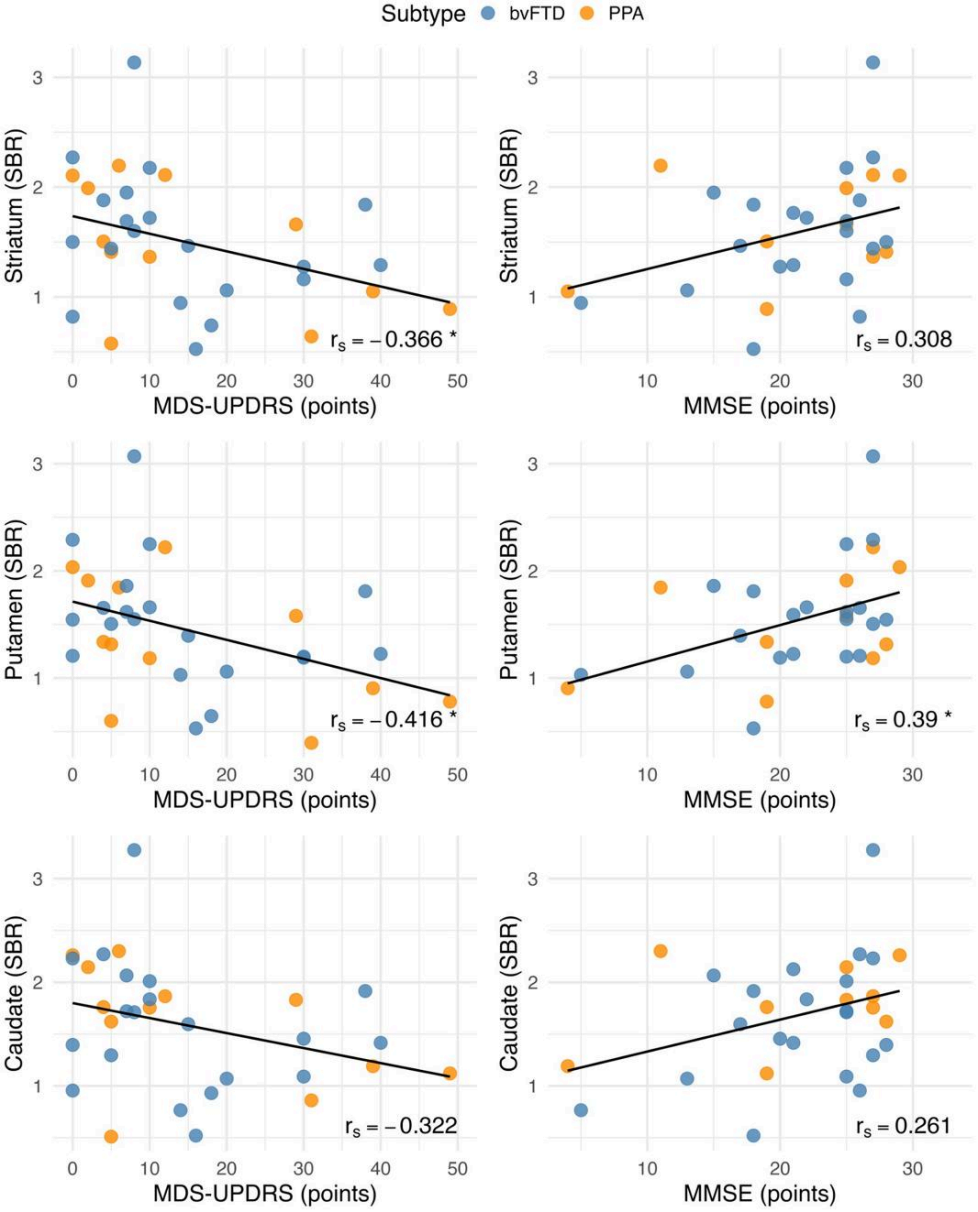
**Associations between specific binding ratios (SBR) of ^123^I-Ioflupane in the striatum, putamen and caudate, and clinical measures.** Scatterplots showing the Spearman partial correlations (*r_s_*) after controlling for age and sex. The linear fit (solid line) is shown. Data points are colour-coded by FTD subtype: bvFTD and PPA. *P < 0.05; **P < 0.01.

In the FTD group, Spearman’s rank correlation coefficient, controlling for age and sex, revealed a negative association between dopamine transporter binding in the striatum (*r_s_* = −0.366, *P* = 0.043, Fig. 3) and the putamen (*r_s_* = −0.416, *P* = 0.02), and Parkinsonian features as measured by MDS-UPDRS. Similarly, dopamine transporter binding in the putamen (*r_s_* = 0.390, *P* = 0.037) was positively correlated with general cognition as measured by MMSE. There was no evidence of a significant correlation between striatal dopamine transporter binding and age (*r_s_* = −0.291, *P* = 0.095), disease duration (*r_s_* = −0.132, *P* = 0.463) or functional impairment evaluated using the CDR global score (*r_s_* = −0.271, *P* = 0.18).

### Correlations of dopamine transporter binding with social cognition

**Figure 4:**
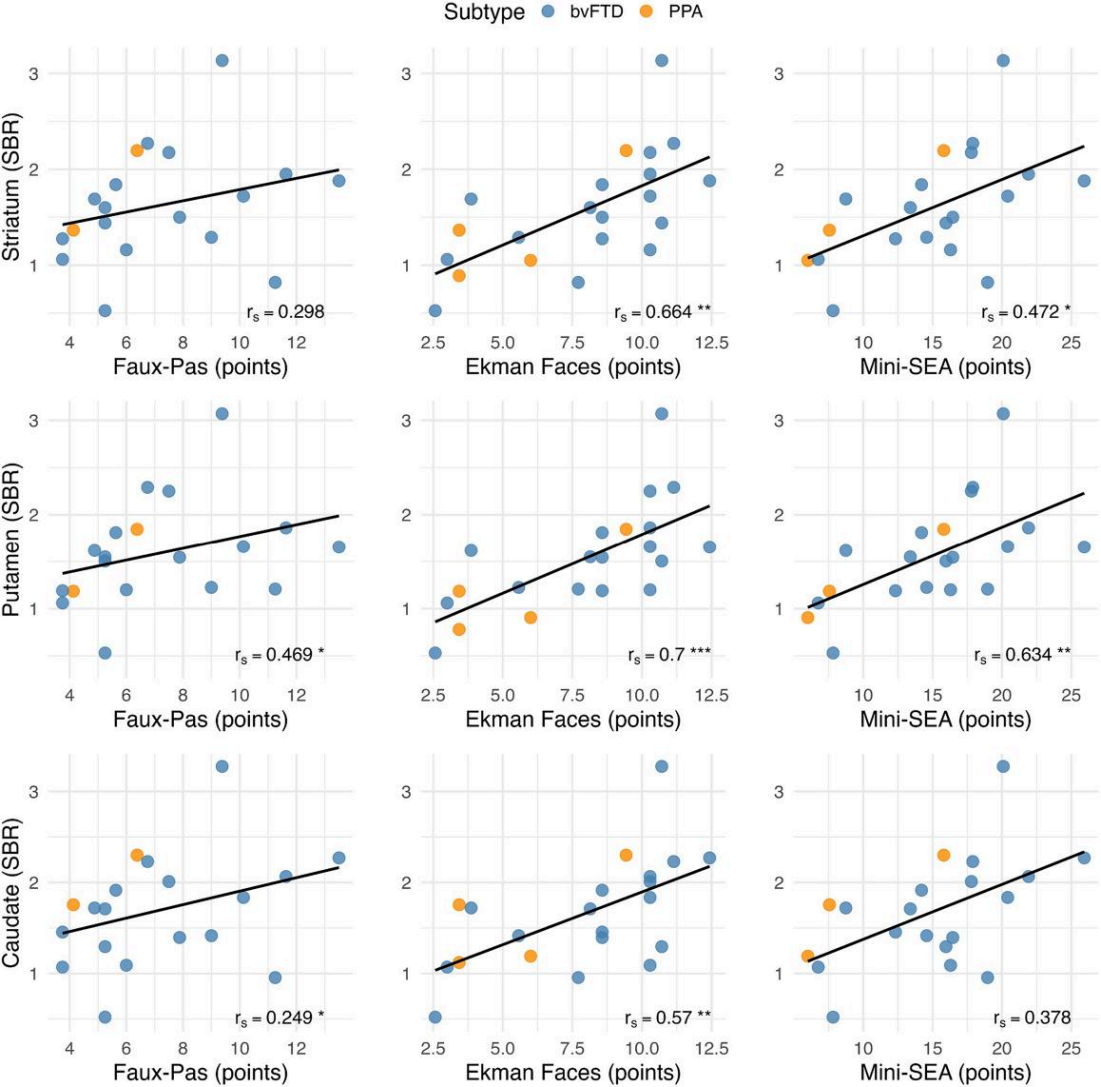
**Associations between specific binding ratios (SBR) of ^123^I-Ioflupane in the striatum, putamen and caudate, and social cognition measures.** Scatterplots showing the Spearman partial correlations (*r_s_*) after controlling for age and sex. The linear fit (solid line) is shown. Data points are colour-coded by FTD subtype: bvFTD and PPA. *P < 0.05; **P < 0.01; ***P < 0.001.

Among patients with FTD, social cognition measured by Mini-SEA correlated with dopamine transporter binding in the striatum (*r_s_* = 0.472, *P* = 0.004, **Table 3,** Fig. 4) and the putamen (*r_s_* = 0.634, *P* = 0.004) using Spearman’s rank correlation coefficient, controlling for age and sex. Regarding the subtests of Mini-SEA, only dopamine transporter binding in the putamen (*r_s_* = 0.269, *P* = 0.049) was positively associated to the Faux-Pas test, while dopamine transporter binding in the striatum (*r_s_* = 0.664, *P* = 0.001), putamen (*r_s_* = 0.700, *P* < 0.001), and caudate (*r_s_* = 0.570, *P* = 0.009) was associated with Facial Emotion Recognition.

**Table 3:** Spearman partial correlations between specific binding ratios of ^123^I-Ioflupane SPECT and social cognition measures in frontotemporal dementia

		Faux-Pas test	Facial emotion recognition	Mini-SEA
Mean striatum	*r_s_*	0.298	0.664	0.472
	*P*	0.230	0.001	0.041
Mean putamen	*r_s_*	0.269	0.700	0.634
	*P*	0.049	<0.001	0.004
Mean caudate	*r_s_*	0.249	0.570	0.378
	*P*	0.320	0.009	0.111

*r_s_* is the Spearman partial correlations after controlling for age and sex. mini-SEA, mini-Social Cognition & Emotional Assessment. mini-SEA comprise the sum of the facial emotion recognition and the short Faux-Pas test scores.

A specific analysis on the different seven emotions of the Facial Emotion Recognition Task showed that the emotions that were most correlated with the measures of dopamine transporter binding were *surprise* (striatum, *r_s_* = 0.664, *P* = 0.001; putamen, *r_s_* = 0.673, *P* = 0.001; caudate *r_s_* = 0.552, *P* = 0.012, Supplementary Table 1), *disgust* (striatum, *r_s_* = 0.590, *P* = 0.019; putamen, *r_s_* = 0.518, *P* = 0.019; caudate *r_s_* = 0.475, *P* = 0.034), and *fear* (striatum, *r_s_* = 0.536, *P* = 0.015; putamen, *r_s_* = 0.463, *P* = 0.010; caudate *r_s_* = 0.478, *P* = 0.033).

### Correlations of dopamine transporter binding with language abilities

We investigated the relationship between language abilities measured by SAND and dopamine transporter binding in the striatum, the putamen, and the caudate using Spearman’s rank correlation coefficient, controlling for age and sex, within the FTD group. (**Table 4**). We found that the measures of dopamine transporter binding were strongly correlated with several language tasks, such as Picture Naming (striatum, *r_s_* = 0.427, *P* = 0.026; putamen, *r_s_* = 0.534, *P* = 0.004; caudate *r_s_* = 0.337, *P* = 0.086), single-word comprehension (striatum, *r_s_* = 0.487, *P* = 0.010; putamen, *r_s_* = 0.531, *P* = 0.004; caudate *r_s_* =0.473, *P* = 0.013), sentence repetition (striatum, *r_s_* = 0.409, *P* = 0.034; putamen, *r_s_* =0.430, *P* = 0.049), semantic associations (striatum, *r_s_* = 0.517, *P* = 0.007; putamen, *r_s_* =0.527, *P* = 0.006; caudate *r_s_* =0.406, *P* = 0.030)¸ and picture description (striatum, *r_s_* = 0.569, *P* = 0.002; putamen, *r_s_* =0.660, *P* < 0.001; caudate *r_s_* = 0.468, *P* = 0.012).

**Table 4:** Spearman partial correlations between specific binding ratios of ^123^I-Ioflupane SPECT and SAND battery items in frontotemporal dementia

		Picture naming	Sentence comprehension	Single-word comprehension	Repetition	Sentence repetition	Reading	Writing	Semantic associations	Picture description
Mean striatum	*r_s_*	0.427	0.245	0.487	0.313	0.409	0.235	0.126	0.517	0.569
	*P*	0.026	0.217	0.010	0.112	0.034	0.229	0.556	0.007	0.002
Mean putamen	*r_s_*	0.534	0.350	0.531	0.313	0.430	0.319	0.278	0.527	0.660
	*P*	0.004	0.074	0.004	0.112	0.049	0.098	0.189	0.006	<0.001
Mean caudate	*r_s_*	0.337	0.189	0.473	0.290	0.290	0.327	0.054	0.406	0.468
	*P*	0.086	0.344	0.013	0.142	0.142	0.237	0.801	0.030	0.012

*r_s_* is the Spearman partial correlations after controlling for age and sex. SAND, Screening for Aphasia in NeuroDegeneration.

### Sensitivity and subgroup analysis

#### Frontotemporal dementia subtypes

No significant differences were found when comparing demographic and clinical characteristics between individuals with bvFTD (*N* = 22) and those with PPA (*N* = 12, Supplementary Table 2). Regarding Dopamine transporter binding using ^123^I-Ioflupane SPECT, no significant differences were found in the striatum, putamen, and caudate binding. However, the bvFTD exhibited a higher putamen-to-caudate ratio compared to patients with PPA (*U* = 5.15, *P* = 0.023).

#### FTD with disease-causing mutations

Individuals with genetic FTD were younger than those with PD (*P* = 0.018) and HC (*P* = 0.049, Supplementary Table 3). Both FTD and PD had lower dopamine transporter binding than HC in the striatum, the putamen, and the caudate (*P* < 0.05). However, there were no differences in striatal binding between FTD and PD (Supplementary Fig. 2). The striatal asymmetry index was significantly different among groups (*H* = 41.5; *P* < 0.001). Post hoc analysis showed that FTD (*P* = 0.008) and PD (*P* < 0.001) had a higher asymmetry index than HC. When comparing genetic (*N* = 7) and sporadic (*N* = 27) FTD, individuals with genetic FTD were younger than those with sporadic FTD (*P* = 0.011). Dopamine transporter binding was comparable between the two groups. However, the putamen-to-caudate ratio was higher in individuals with genetic FTD (*P* = 0.031). Finally, to assess the potential influence of genetic cases, we conducted a sensitivity analysis excluding the seven individuals with genetically confirmed FTD. The results remained consistent, with all group differences between sporadic FTD and healthy controls remaining highly significant (*P *< 0.001) across all striatal regions.

## Discussion

In this study, we demonstrated that dopamine transporter (DAT) levels are significantly reduced in FTD compared to controls, with a symmetrical loss observed in both the putamen and the caudate, particularly in patients with genetically confirmed disease. Additionally, our analysis highlighted significant associations between DAT levels and core clinical features of FTD, including general cognition, social cognition (particularly facial emotion recognition) and language abilities.

We have demonstrated that ^123^I-Ioflupane SPECT, a widely available dopamine transporter imaging modality, can detect lower dopamine transporter levels in patients with FTD compared to age- and sex-matched controls, although higher than in patients with PD. These results are in line with reports from smaller sample sizes that support the impairment of the dopaminergic system in FTD.^17,18^ Rinne *et al.* using PET and ^[11C]^CFT showed a reduction of tracer binding in the caudate nucleus and putamen of 12 patients with FTD. Similarly, Sedaghat *et al.* demonstrated that the uptake of ^123^I-Ioflupane in the right and left striatum of seven patients with FTD was reduced to 62% and 68%, respectively, compared to controls. Moreover, consistent with our study, the motor UPDRS score of the patients with FTD showed a negative correlation to the uptake of the radiotracer, suggesting that parkinsonian symptoms are associated with striatal dopaminergic denervation, especially of the putamen. Another study on the diagnostic accuracy of ^123^I-Ioflupane SPECT for the differentiation of FTD from dementia with Lewy bodies (DLB)^21^ showed that the uptake of striatal DAT is reduced in some cases of FTD and that ^123^I-Ioflupane SPECT is less useful in differentiating FTD from DLB than it is in differentiating AD from DLB. Similarly, in our study, in which the mean MDS-UPDRS score was about 14, in the study by Morgan *et al*. the overall frequency of parkinsonian features was surprisingly high in FTD and reached similar levels to that observed in DLB. Although parkinsonian motor features were originally included as a supportive feature in the *Neary* clinical criteria,^53^ they were subsequently excluded from later revisions,^4^ even though numerous studies have reported high prevalence in FTD.^17,18,20^ Additional support for the dysregulation of the basal ganglia in FTD is derived from structural imaging^22-25,27,54-56^ and the pathological modifications identified in the striatum and substantia nigra in postmortem studies.^26,57-59^ In a neuropathological study involving individuals with transactive response DNA-binding protein-43 kDa (TDP-43)-related frontotemporal lobar degeneration (FTLD-TDP), phosphorylated TDP-43 accumulation was found in the neurons of striatal efferent pathways, their corresponding tracts, or axon terminals located in the substantia nigra pars reticulata (SNr) and globus pallidus (GP).^26^ This suggests that striatal efferent projections are often involved in TDP-43-related FTD and may influence its clinical manifestations.

A large coordinate-based meta-analysis of structural and functional brain studies on bvFTD revealed consistent alterations in the striatum, especially in functional studies of bvFTD patients versus healthy controls.^22^ This suggested a key involvement of the salience network and subcortical regions in the pathophysiology of bvFTD. Another recent study investigated the long-term anatomical progression of the three FTD variants using MRI staging schemes and described the sequential divergence of volumetric trajectories between normal aging and FTD.^27^ They reported that striatal atrophy precedes focal cortical atrophy with a ‘radiological’ prodromal phase lasting 8–10 years. This implies that striatal atrophy could potentially serve as a biomarker for identifying preclinical or prodromal FTD variants. Whether or not dopamine transporter imaging can also serve as an early marker for preclinical/prodromal FTD variants remains to be investigated.

In this study, we observed that both the putamen and the caudate were similarly affected in FTD, reflecting a more uniform distribution of dopaminergic loss across the striatum, in contrast to the posterior-to-anterior gradient typically observed in patients with early Parkinson’s disease.^32^ Some recent case reports have suggested that dopamine transporter levels are decreased, especially in the caudate of patients with FTD. In 2020, Liu *et al.* reported a case of frontotemporal lobar degeneration–motor neuron disease (FTLD-MND), with a heterozygous pathogenic variant of the *TBK1* gene (p.E463del) severe loss of dopamine transporter binding in the basal ganglia, with an almost total loss in the caudate nucleus, providing direct evidence of severe dopaminergic damage in the caudate.^28^ Subsequently, other reports showed dominantly affected caudate nucleus with relatively preserved dopamine transporter activity in the putamen of patients with sporadic^31^ and familial FTD with *C9orf72* repeat expansions.^29^ Of note, also patients with disease-causing FTD mutations (including four *C9orf72* and three *GRN*) showed a relatively uniform pattern of striatal dopaminergic loss. Interestingly, *fused in sarcoma* (FUS), one of the key proteins implicated in FTLD-FET,^60^ is associated with early and prominent caudate atrophy.^61^ Some authors have recently proposed that the involvement of the caudate nucleus and its network in FTLD-FET may be the upstream mechanism of neurodegeneration and the driver of cognitive impairment.^62^

Our data indicate that the levels of dopamine transporter in the striatum are related to general cognition, as assessed by MMSE, and to social cognition, especially theory of mind and facial recognition tasks. These findings are consistent with previous MRI studies that suggested a link between behavioural impairment and striatal atrophy in FTD.^23,24,63-65^ Voxel-based morphometry analyses showed that anhedonia was linked to reduced volume in a wide frontostriatal network, comprising orbitofrontal and medial prefrontal, paracingulate and insular cortices, as well as the putamen.^63^ Moreover, a study found that cortical thickness in caudal, lateral and superior frontal regions and caudate nucleus volume were inversely correlated with apathy severity scores of the Neuropsychiatric Inventory Questionnaire (NPI).^64^ Likewise, another study using manual Region of Interest tracing for volume quantification found that putamen volume also correlated significantly with NPI score.^23^ Additionally, another study revealed that behavioural symptoms and severity of bvFTD were associated with abnormalities in striatal size and shape.^24^ Furthermore, recent structural imaging studies have also provided evidence that social cognition in FTD may be related to striatal atrophy. Voxel-based morphometry was employed to identify patterns of grey matter loss associated with task performance of social norm compliance in 22 patients with bvFTD.^66^ In the prosocial condition, patients’ performance indicated a diminished expression of prosocial behaviour, related to reduced grey matter in the anterior insula, lateral orbitofrontal cortex, anterior cingulate and dorsal striatum. This suggests that the striatum may have a unique role in computing social reward and influencing action-output oriented to seek rewarding social interactions.^66^ A large cohort study of familial FTD patients carrying mutations in *C9orf72*, *GRN*, or *MAPT* genes from the GENFI multicentre cohort revealed that social cognition impairment, assessed by Facial Emotion Recognition and Faux-Pas Recognition tests, was mainly related to a network of left-hemisphere regions, especially the striatum, orbitofrontal cortex, and insula.^67^ Likewise, empathy, evaluated by informant-based Interpersonal Reactivity Index scores, decreased from asymptomatic to very mild symptomatic stages in both genetic and sporadic bvFTD, regardless of the pathogenic variant, and was associated with subcortical atrophy in the left caudate.^68^ Another noteworthy study from the GENFI initiative employed cross-modal correlation of MRI-based measures with nuclear imaging–derived estimates of various neurotransmitter systems, indirectly assessing neurotransmitter deficits in monogenic FTD.^69^ The study found that social cognition scores, loss of empathy, and poor response to emotional cues correlated with the strength of grey matter volume colocalization of dopamine and serotonin pathways. By demonstrating the association between dopamine transporter levels in the striatum and social cognition, we bolster the emerging framework that considers symptoms of FTD as stemming from impaired goal-directed social behaviour,^15^ which may depend on dopaminergic dysfunction in the striatum.

We also found that dopamine transporter levels in the striatum correlated with linguistic impairment in FTD, opening new avenues to investigate the relationships between the dopaminergic system and the clinical manifestations of aphasia in FTD. Although one study found a relationship between anomia and striatal volumes in *GRN* mutation carriers,^70^ this is the first study, to the best of our knowledge, that finds a direct relationship between dopaminergic imaging in the striatum and language abilities in FTD. A previous study, which indirectly assessed neurotransmitter deficits in PPA,^71^ showed that voxel-based brain changes in PPA were significantly associated with the spatial distribution of serotonin, dopamine, and glutamatergic pathways and that disease severity was negatively correlated with the strength of grey matter volumes colocalization of dopamine receptor D1. Dopaminergic agents have been employed in post-stroke aphasia, but the overall evidence for their efficacy is mixed.^72^ It is worth noting that, apart from bromocriptine and levodopa, there is a scarcity of dopaminergic agents that have been tested and that the trials involving patients with PPA are very limited.^72^

Dopaminergic therapies have been investigated in FTD, although available evidence remains limited and primarily focused on symptomatic management.^73^ Dopaminergic stimulants such as methylphenidate^74^ and dextroamphetamine^75^ have shown some promise in alleviating behavioural disturbances, particularly apathy and impulsivity, in small randomized trials of patients with bvFTD. However, dopamine replacement therapies have generally shown minimal benefit for parkinsonian features in FTD, with only isolated case reports suggesting clinical improvement.^76^ In the context of PPA, a small crossover trial of the D2 agonist bromocriptine (22.5 mg/day) in six patients with the non-fluent variant reported limited benefit, including modest improvement in utterance length.^72,77^ Notably, an ongoing Phase IIa randomized controlled trial (NCT04937452) is currently evaluating the non-ergot dopamine agonist rotigotine (4–6 mg/24 h, transdermal) in 75 patients with bvFTD over a 24-week period. This trial aims to assess the drug’s impact on behavioural symptoms and frontal lobe metabolism as measured by FDG-PET, and may provide valuable insight into the therapeutic modulation of dopaminergic tone in FTD.

One limitation of our study is that we used public data from an international dataset for the striatal binding ratios of healthy controls. However, the data from this dataset (PPMI) have been widely validated^78,79^ and the striatal binding ratios of healthy controls are consistent with those of reference studies^18,19^ and with those of the European multicentre database of healthy controls for ^123^I-Ioflupane SPECT.^45^ Another limitation is the focus on the dopamine transporter levels in the putamen and caudate, which may not capture the whole spectrum of dopaminergic dysfunction in FTD. Future studies could explore extra-striatal dopamine transporter uptake using ^123^I-Ioflupane SPECT^80^ and other imaging and tracer modalities. Another limitation is that structural brain changes in FTD, such as caudate atrophy or frontal horn widening, may partially contribute to the observed reduction in dopamine transporter binding. Future studies should incorporate partial volume correction techniques to more accurately assess dopaminergic function in the context of neurodegeneration. We also acknowledge that the use of the original CDR, rather than the FTLD-modified version, may have reduced sensitivity to detect functional impairment in domains typically affected in FTD, and might have contributed to the lack of clear associations between SBR and CDR scores observed in this study.

A major strength of our study is the inclusion of patients with genetically confirmed FTD, which increases the reliability and specificity of our findings. We found that the degree of dopaminergic denervation was even more pronounced in patients with genetic FTD than in those with sporadic FTD. Another strength is the comparison of striatal dopaminergic distribution patterns across FTD, PD, and healthy controls, which highlights that the pattern of dopaminergic dysfunction in FTD, characterized by a more uniform involvement of the putamen and caudate, differs from the posterior-to-anterior gradient typically observed in synucleinopathies. This suggests that dopamine transporter imaging could be useful to differentiate between these neurodegenerative disorders. Moreover, the fact that the clinical and dopaminergic imaging characteristics were similar between bvFTD and PPA patients makes our study more significant, showing that dopamine transporter imaging is a marker of the FTD spectrum and correlates with the core features of language and social cognition dysfunction. This supports the notion that FTD is a heterogeneous syndrome with a common underlying pathology.

In conclusion, we demonstrated that dopamine transporter levels are lower in FTD than in healthy subjects, and that striatal dopaminergic loss in FTD shows a more uniform distribution across the putamen and caudate, differing from the posterior-to-anterior gradient typically observed in Parkinson’s disease. We also revealed that dopamine transporter levels are associated with parkinsonian symptoms and cognitive performance in FTD. Moreover, we found that social cognition and language impairment correlated with dopamine transporter levels in both the putamen and the caudate. These results indicate that the pattern of dopamine transporter levels could be a useful biomarker of FTD and pave the way for further studies on the role of the dopaminergic system and the striatum in the cardinal clinical features of FTD.

## Supplementary Material

fcaf284_Supplementary_Data

## Data Availability

The ethical requirement to ensure patient confidentiality precludes public archiving of our data. Researchers who wish to obtain the raw data should reach out to the corresponding author, who will coordinate with the ethics committee that authorized the study. Based on their approval, we will provide the necessary data to replicate the findings to the individual researcher.
