# Supplementary material for: Striatal dopaminergic patterns and clinical features in frontotemporal dementia
Source: Brain Commun. 2025 Aug 20;7(4):fcaf284. doi: 10.1093/braincomms/fcaf284 (PMC12365758; doi:10.1093/braincomms/fcaf284)
Supplement: fcaf284_Supplementary_Data [file fcaf284_supplementary_data.pdf]

# Supplementary Material

## Language Assessment

The SAND is a screening tool capable of identifying essential linguistic features crucial for diagnosing and categorizing PPA. It encompasses nine assessments: picture naming, sentence comprehension, single-word understanding, word and non-word repetition, sentence repetition, reading, writing, semantic association, and picture description. For this study, we solely utilized quantitative outcomes, excluding qualitative measures.

*Picture Naming*: Participants were asked to name 14 black and white drawings, each displayed for 6 seconds. The images comprised seven living and seven non-living items, allowing differentiation between these categories during scoring (living item score range: 0–7; non-living item score range: 0–7). A point was awarded for each correct response, 0.5 points for correct responses given after a phonological cue, and 0 for incorrect or missed responses. Additionally, error types (distortions, phonological, visual, and semantic errors) were reported to allow qualitative assessment of performance.

*Auditory Sentence Comprehension*: Participants encountered eight sentences with varying syntactic structures. They were required to select the picture that corresponded to the sentence read by the examiner. One point was assigned for a correct answer and 0 for an incorrect response. Qualitative assessment distinguished errors in selecting morphological and thematic distractors.

*Single-Word Comprehension:* Four pictures were presented per trial, one corresponding to the spoken word. The test comprised twelve trials, six involving living and six non-living pictures, allowing independent scoring for both categories (living item score range: 0–6; non-living item score range: 0–6). A point was given for correct answers, 0 for incorrect responses.

*Words and Non-Words Repetition:* Participants repeated ten items, including six words and four non-words read by the examiner. Each correct response earned one point, and 0 was given for an incorrect answer. A qualitative assessment noted distortions, phonological, morphological, semantic, and lexicalization errors, as well as omissions.

*Sentence Repetition*: Six sentences were read aloud to participants, three predictable and three unpredictable. They were asked to repeat the sentences. One point was awarded for a correct answer and 0 for an incorrect response. Error types (distortions, phonological, morphological, and semantic errors) and omissions were reported for qualitative analysis.

*Reading*: Participants were tasked with reading sixteen items: twelve words and four non-words. A point was given for each correct answer, and 0 for an incorrect response. Additionally, qualitative analysis detailed distortions, phonological, semantic, and morphological errors, regularizations, lexicalization errors, and omissions.

*Writing*: The writing test tasked individuals with describing how to brush teeth. Quantitative analysis included Information Units and six language features, with qualitative analysis covering various errors and modifications.

*Semantic Association*: Participants were presented with four trials, each consisting of three images, and asked to identify two semantically related images. One point was given for a correct answer, 0 for an incorrect response.

Picture description: Participants described a seaside scene, assessed quantitatively through Information Units and eight language features, with qualitative analysis of errors, modifications, and speech flow aspects.

## Representativeness of the Selected Control Subgroup

To assess the representativeness of the healthy control participants included in this study, we conducted a supplementary analysis using the centrally processed specific binding ratio (SBR) values from the Parkinson's Progression Markers Initiative (PPMI). We compared the 37 controls included in our analyses with the remaining 159 eligible PPMI controls. This comparison revealed no statistically significant differences in mean SBR values in the striatum ($p = 0.253$), putamen ($p = 0.239$), or caudate ($p = 0.300$). These findings support the representativeness of our selected control subgroup in terms of dopaminergic binding profiles.

**Supplementary Table 1. Spearman partial correlations between specific binding ratios of 123I-Ioflupane SPECT and Ekman Faces test scales subitems in Frontotemporal Dementia.**

| | | Anger | Happiness | Disgust | Sadness | Fear | Surprise | Neutral |
|---|---|---|---|---|---|---|---|---|
| **Mean Striatum** | $r_s$<br>*p* | 0.249<br>0.291 | 0.278<br>0.236 | 0.520<br>0.019 | 0.165<br>0.487 | 0.536<br>0.015 | 0.664<br>0.001 | 0.440<br>0.052 |
| **Mean Putamen** | $r_s$<br>*p* | 0.393<br>0.086 | 0.398<br>0.082 | 0.518<br>0.019 | 0.154<br>0.518 | 0.463<br>0.010 | 0.673<br>0.001 | 0.533<br>0.016 |
| **Mean Caudate** | $r_s$<br>*p* | 0.223<br>0.344 | 0.187<br>0.430 | 0.475<br>0.034 | 0.099<br>0.679 | 0.478<br>0.033 | 0.552<br>0.012 | 0.381<br>0.097 |

$r_s$ is the Spearman partial correlations after controlling for age and sex

**Supplementary Table 2. Demographic, clinical characteristics and Specific Binding Ratios of Frontotemporal Dementia, behavioural variant and Primary Progressive Aphasia patients**

| | Behavioural Variant FTD | Primary Progressive Aphasia | Test Statistic |
|---|---|---|---|
| **Demographics** | | | |
| ***n*** | 22 | 12 | |
| **Age, years** | 66.36 (9.99) | 66.92 (9.39) | *U* = 0.1, *p*=0.942 |
| **Sex (male/female)** | 13/9 | 10/2 | $\chi^2$ = 0.1, *p*=0.289 |
| **Disease Duration, years** | 4.09 (2.58) | 2.83 (1.27) | *U* =1.84, *p*=0.175 |
| **Global** | | | |
| **CDR Global score** | 1.13 (0.57) | 1.06 (0.62) | *U* =1.5, *p*=0.690 |
| **MMSE** | 21.55 (5.88) | 21.40 (8.25) | *U* =0.2, *p*=0.610 |
| **MoCA** | 19.44 (4.26) | 9.00 (5.10) | *U* =7.8, *p*=0.005 |
| **MDS-UPDRS III** | 12.75 (12.41) | 16.00 (16.52) | *U* = 0.30, *p*=0.861 |
| **Specific Binding Ratios** | | | |
| **Mean Striatum** | 1.51 (0.60) | 1.46 (0.58) | *U* = 0.1, *p*=0.971 |
| **Mean Putamen** | 1.49 (0.57) | 1.34 (0.59) | *U* = 0.2, *p*=0.588 |
| **Mean Caudate** | 1.57 (0.64) | 1.60 (0.57) | *U* =0.2, *p*=0.614 |
| **Asymmetry Ratio** | 17.34 (21.72) | 13.8 (21.59) | *U* =0.49, *p*=0.662 |
| **Putamen-to- caudate ratio** | 0.97 (0.17) | 0.83 (0.20) | *U* =5.15, *p*=0.023 |

Mean (standard deviation) scores are shown unless otherwise indicated. FTD, Frontotemporal Dementia; CDR, Clinical Dementia Rating Scale; MMSE, Mini-Mental State Examination; MoCA, Montreal Cognitive Assessment; MDS-UPDRS, Movement Disorders Society Unified Parkinson's Disease Rating Scale. Specific Binding Ratios refers to 123I-Ioflupane SPECT specific bindings in the striatum, putamen and caudate.

**Supplementary Table 3. Demographic and clinical characteristics of genetic Frontotemporal Dementia, Parkinson's disease patients and healthy controls.**

| | Frontotemporal Dementia | Parkinson's Disease | Healthy Controls | Test Statistic |
|---|---|---|---|---|
| **Demographics** | | | | |
| ***n*** | 7 | 68 | 37 | |
| **Age, years** | 58.00 (9.09) | 68.65 (10.95) | 66.52 (5.34) | $H$ = 11.8, $p$ = 0.008 |
| **Sex (male/female)** | 3/4 | 48/20 | 25/12 | $\chi^2$ = 2.2, $p$=0.325 |
| **Disease Duration, years** | 4.00 (2.45)[a] | 5.25 (3.90)[c] | NA | $U$ = 0.513, $p$=0.473 |
| **Global** | | | | |
| **CDR Global score** | 1.00 (0.61)[a] | 0.04 (0.17)[c] | NA | $U$ =35.5, $p$<0.001 |
| **MMSE** | 23.00 (5.05)[a] | 25.60 (4.14)[c] | NA | $U$ =1.7, $p$=0.192 |
| **MoCA** | 19.50 (6.35) | NA | 28.38 (1.04) | $U$ = 11.4, $p$=<0.001 |
| **MDS-UPDRS III** | 5.67 (5.61)[a,b] | 30.59 (12.65) [a,c] | 1.05 (1.47) | $H$ = 81.2, $p$=<0.001 |
| **Specific Binding Ratios** | | | | |
| **Mean Striatum** | 1.38 (0.99) [a,b] | 0.96 (0.49) [a,c] | 2.27 (0.47) | $H$ = 73.8, $p$=<0.001 |
| **Mean Putamen** | 1.42 (0.94) [a,b] | 0.84 (0.45) [a,c] | 2.30 (0.48) | $H$ = 100.2, $p$=<0.001 |
| **Mean Caudate** | 1.42 (1.03) [a,b] | 1.25 (0.56) [a,c] | 2.39 (0.54) | $H$ = 46.3, $p$=<0.001 |
| **Asymmetry Ratio** | 32.33 (30.33)[a] | 24.72 (32.57)[a] | 3.70 (3.01) | $H$ =41.5, $p$<0.001 |
| **Putamen-to-caudate ratio** | 1.04 (0.13) [b] | 0.68 (0.20)[a,c] | 0.96 (0.03) | $H$ =58.2, $p$<0.001 |

*Mean (standard deviation) scores are shown unless otherwise indicated; a, different from health control at <0.05.; b, different from Parkinson's Disease at <0.05; c, different from Frontotemporal Dementia at <0.05. CDR, Clinical Dementia Rating Scale; MMSE, Mini-Mental State Examination; MoCA, Montreal Cognitive Assessment; MDS-UPDRS, Movement Disorders Society Unified Parkinson's Disease Rating Scale*

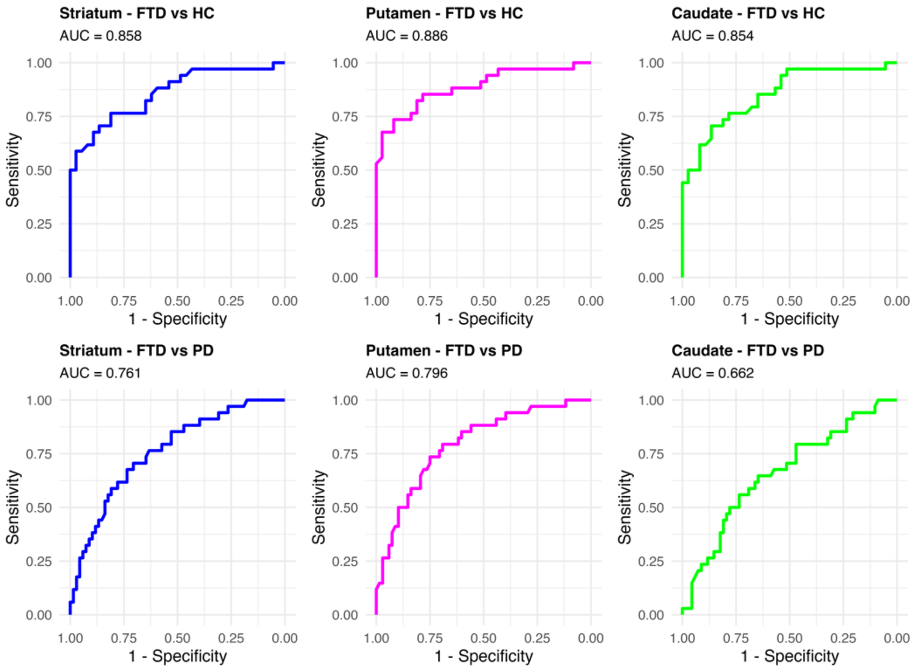


**Supplementary Figure 1. Diagnostic accuracy of dopamine transporter binding ratios in FTD.** Receiver Operating Characteristic (ROC) curves displaying the diagnostic performance of dopamine transporter specific binding ratios (SBR) in the striatum (left column), putamen (middle column), and caudate (right column) for discriminating frontotemporal dementia (FTD) from healthy controls (HC, top row) and Parkinson's disease (PD, bottom row). The Area Under the Curve (AUC) values are shown in each panel. Higher AUC values indicate better discrimination.

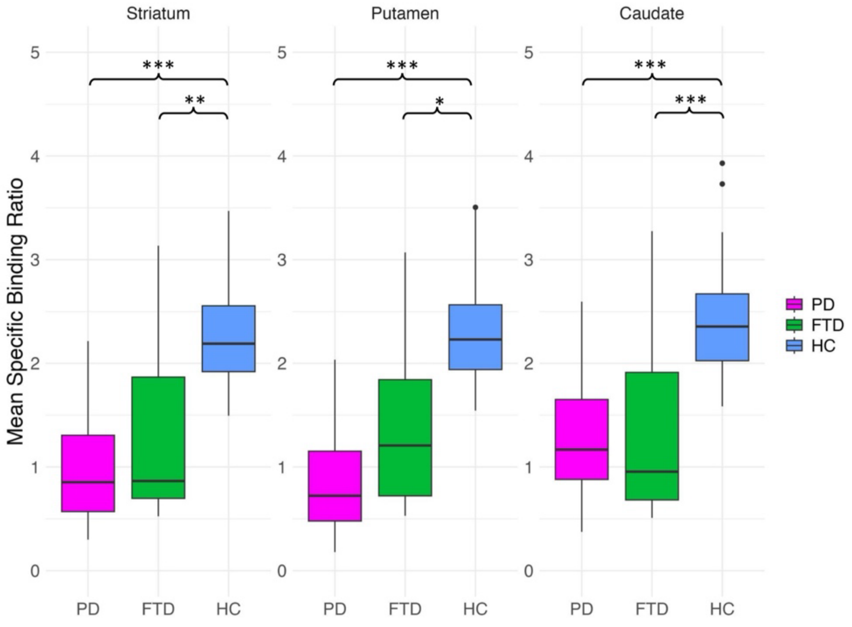


**Supplementary Figure 2. Dopamine transporter binding in genetic FTD, PD and controls.** Box and whisker plots displaying the distribution of specific binding ratios of $^{123}$I-Ioflupane among the Parkinson's Disease (PD, n=68, magenta), genetic Frontotemporal Dementia (FTD, n=7, green), and Healthy Controls (HC, n=37, blue) groups. The box represents the interquartile range (IQR; 25–75th percentile) with the median value (horizontal line) inside. The whiskers extend from the box to the minimum and maximum values that are not outliers, and the outliers are shown as dots. Outliers are defined as values outside the range [Q1 - 1.5 * IQR, Q3 + 1.5 * IQR], where Q1 is the 25th percentile and Q3 is the 75th percentile. Statistical analysis was conducted using the Kruskal–Wallis test for group comparison, followed by Mann–Whitney U tests with Bonferroni correction for post hoc pairwise comparisons. *$P$-value < 0.05, *** $P$-value < 0.001.
